# The Epidemiology and Geographic Distribution of Nontuberculous Mycobacteria Clinical Isolates from Sputum Samples in the Eastern Region of China

**DOI:** 10.1371/journal.pntd.0003623

**Published:** 2015-03-16

**Authors:** Yan Shao, Cheng Chen, Honghuan Song, Guoli Li, Qiao Liu, Yan Li, Limei Zhu, Leonardo Martinez, Wei Lu

**Affiliations:** 1 Department of Chronic Communicable Disease, Center for Disease Control and Prevention of Jiangsu Province, Nanjing, China; 2 Department of Epidemiology and Biostatistics, University of Georgia School of Public Health, Athens, Georgia, United States of America; Fondation Raoul Follereau, FRANCE

## Abstract

**Background:**

Nontuberculous mycobacteria (NTM) have been reported to be increasing worldwide and its geographic distribution differs by region. The aim of this study was to describe the epidemiology and distribution of NTM in the eastern part of China.

**Methods:**

Sputum samples were collected from 30 surveillance sites for tuberculosis drug resistance test from May 1, 2008 to December 31, 2008. Identification was performed using a biochemical test, multiplex PCR and GenoType Mycobacterium CM/AS assay.

**Results:**

A total of 1779 smear positive clinical isolates were obtained, of which 60 (3.37%) were NTM. Five species/complex of NTM were identified; *M*. *intracellulare* was the predominated species (68.33%), followed by *M*. *abscessus-M*. *immunogenum* (13.33%), *Mycobacterium spec*. (10.00%), *M*. *Kansasii* (6.67%) and *M*. *peregrinum-M*. *alvei-M*. *septicum* (1.67%).

**Conclusion:**

*M*. *intracellulare* was the main species of NTM in the eastern part of China and clinical physicians should pay more attention to NTM induced pulmonary disease.

## Author Summary

Nontuberculous mycobacteria (NTM) exist ubiquitously in the environment and cause many kinds of diseases including pulmonary infection. Despite this, NTM does not match compulsory report policy in many countries, such as China. Thus, the epidemiology of NTM is generally unknown. Furthermore, misdiagnosis of nontuberculous mycobacterium disease as multi-drug resistant tuberculosis (MDR-TB) frequently occurs in clinical settings because of similar clinical manifestations. Therefore, elucidating the epidemiology and distribution of NTM species is important and may have a profound and lasting impact on the prevalence of pulmonary NTM disease. In our study, we enrolled smear-positive sputum samples during 2008 from Jiangsu province in the eastern region of China. Traditional biochemical tests and molecular biological methods were performed to distinguish NTM isolates to species/complex level. For the first time, we provide a snapshot of the epidemiology and geographic distribution of NTM in Jiangsu province. The proportion of NTM was 3.37% of all the *Mycobacterium* isolates and the species of NTM differed by area.

## Introduction

Nontuberculous mycobacteria (NTM) were observed soon after Koch’s discovery of Mycobacterium tuberculosis [1]. However, only until the 1950s NTM were defined as ‘atypical’ or ‘anonymous’ mycobacteria [2]. It is well known that more than 100 species of NTM are ubiquitously distributed in the environment, fresh and salt water, soil and biofilms [3,4,5].

Pulmonary disease caused by NTM has gained increased attention in the world and several studies indicate that NTM incidence is increasing [6,7,8]. Kozo Morimoto et al. estimated that the prevalence rate of pulmonary disease caused by NTM was 33–65 per 100,000 [9]. Meanwhile, due to inappropriate treatment and high treatment failure [10,11], the mortality of NTM caused lung disease was high at around 30% [12]. Therefore, efficient detection and regular monitoring of NTM is crucial. However, reporting NTM disease to the government is not compulsory according to the infectious disease control policy in China [13]. Thus, knowledge about the epidemiology and distribution of NTM causing pulmonary disease is limited in China, especially in the countryside.

Several studies have shown that different NTM species exhibit varied pathogenicity and have different antibiotic susceptibility patterns [14,15]. Meanwhile, it is often seen that pulmonary disease caused by NTM were misdiagnosed as multi-drug resistant tuberculosis (MDR-TB), especially in the developing countries with a high burden of *M*. *tuberculosis* disease [16]. Because in such countries, most pulmonary symptoms resembling mycobacterial disease is presumed as *M*. *tuberculosis*, but NTM is often resistant to first-line anti-TB drugs, subsequently treated for multidrug resistant (MDR) disease. Our study will also establish proper assay procedure for improving the diagnostic accuracy for NTM caused lung disease.

Conventional identification of NTM at the species level is primarily based on phenotypic characteristics as biochemical tests are not only time-consuming but also error prone [17]. However, the molecular biological method has been applied more commonly, and it facilitates the detection of NTM from clinical samples. Amplification of the 16S rRNA was chosen to provide the positive control when evaluating Mycobacteria by PCR and Rv0577 was a genotypic marker for the *M*. *tuberculosis* complex (MtbC) [18]. In our study, we chose both of them to differentiate MtbC from Mycobacteria other than MtbC species (MOTT). Besides that, recently DNA strip assays for the identification of Mycobacteria to the species level have been developed, GenoType Mycobacterium CM/AS assay (Hain Lifescience, Nehren, Germany) is one of them. This assay is based on reverse hybridization of a PCR product to a nitrocellulose strip with immobilized probes for different mycobacterial species and shows high concordance with 16S rRNA and biochemical tests [19]. We performed this assay to differentiate NTM species of the samples from tuberculosis suspicious patients to assist clinical diagnosis.

## Methods

### Sample collection

Sputum samples were consecutively collected from 30 tuberculosis drug resistance surveillance sites in Jiangsu province, China, during May 1, 2008 to December 31, 2008. Only patients with suspicious tuberculosis symptoms, such as cough for at least 2 weeks and abnormal chest X-ray manifestation, were recruited. All samples were derived from the lungs and at least one of three samples per patient were smear positive by the Ziehl-Neelsen method. Then, two of the three samples were chosen to be inoculated on the Löwenstein-Jensen (LJ) medium for culture. Finally, a total of 1779 clinical isolates were obtained. All samples collected were anonymized. This study was approved by the Institute Ethics Committee of Jiangsu Provincial Center for Disease Control and Prevention.

### Mycobacterium DNA preparation

DNA from mycobacterial culture was extracted following procedure. For each sample, one loop of cultures was suspended in 400ul TE buffer, boiled at 95°C for 30 minutes, then followed by ice-bath for 5 minutes and centrifugation at 12000×g for 5 minutes. Finally, 200μl of the DNA supernatant was used for further testing, while the remainder was stored at −20°C.

### Identification of NTM

As a preliminary screening, p-nitrobenzoic acid (PNB) and thiophene carboxylic acid hydrazine (TCH) was used for NTM identification at first.

Growth on LJ medium containing PNB indicates that the bacilli do not belong to MtbC. In order to distinguish MOTT from MtbC, all of the MtbC isolates identified by PNB were tested by 16S rRNA and Rv0557 again. Finally, we used GenoType Mycobacterium CM/AS assay for further identification to species/complex level. The GenoType Mycobacterium CM/AS assay was performed according to the instructions of the manufacturer.

## Results

**Fig 1:**
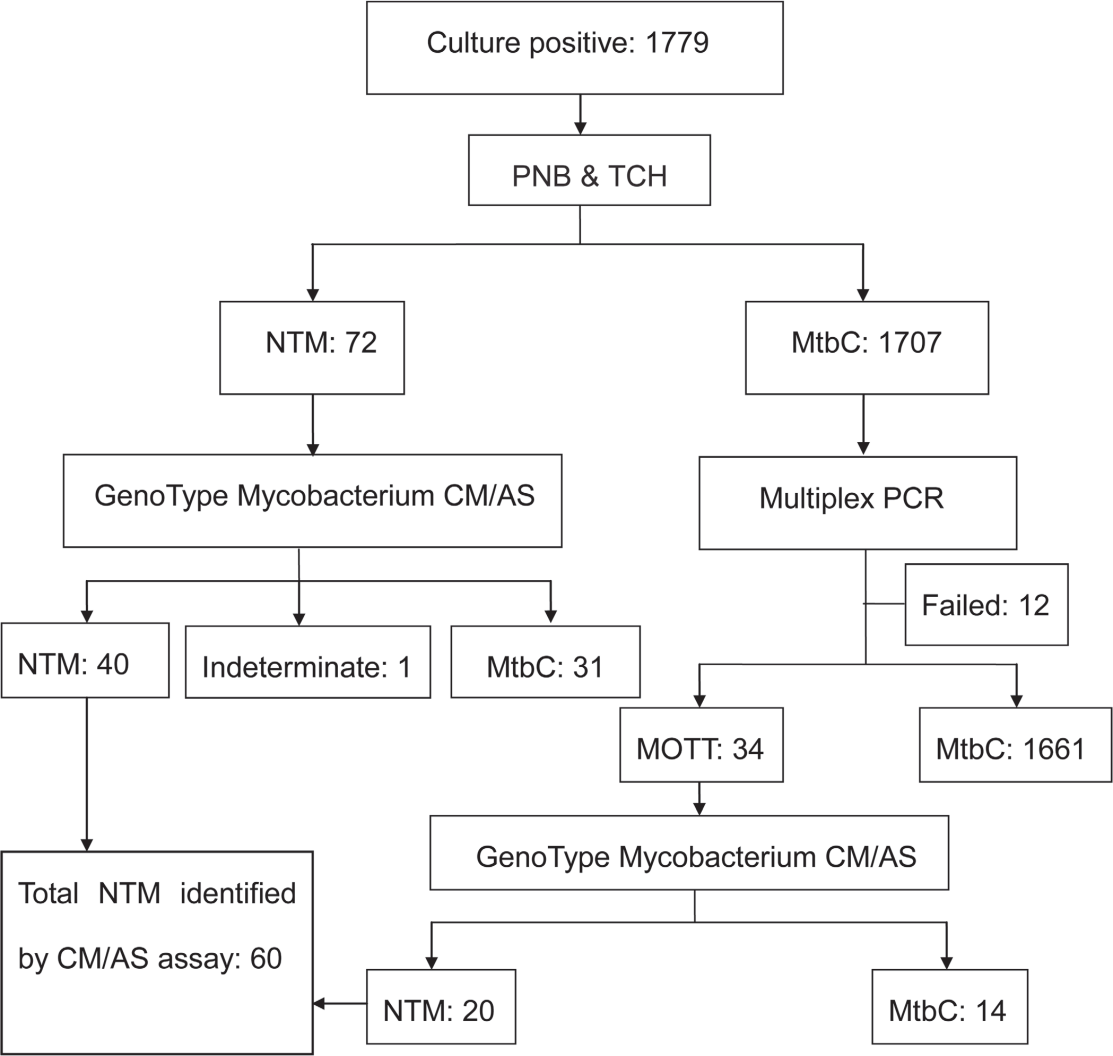
Flow chart of the NTM screening procedure. Abbreviations: PNB: P-nitrobenzoic acid; TCH: Thiophene carboxylic acid hydrazine; NTM: Non-tuberculosis Mycobacteria; MtbC: *M*. *tuberculosis* complex; MOTT: Mycobacteria other than Mycobacterium tuberculosis complex.

We collected 1779 positive cultures, from May 1, 2008 to December 31, 2008. The flow chart of NTM identification was shown in Fig. 1. After screening by PNB and TCH resistant test, 72 samples were classified as NTM and 1707 samples belonged to the MtbC. For those MtbC samples determined by PNB and TCH method, multiplex PCR of 16S rRNA and Rv0557 was carried out for confirmation. Finally, we obtained 106 strains including NTM (n = 72) and MOTT (n = 34) to perform GenoType Mycobacterium CM/AS assay.

The CM/AS assay is based on a multiplex PCR targeting species-specific DNA regions combined with a reverse hybridization format (DNA strip). The specific patterns are composed of obligatory and additional facultative stainings that can be visually identified by clear-cut hybridization signals on the membrane strips. After an interpretation, sixty out of 106 strains were identified to species/complex level, forty five MtbC strains and one strain showed non species-specific lines were excluded (Fig. 1). Therefore, the rate of NTM was 3.37% (60/1779) in Jiangsu province.

The band patterns of all NTM determined by GenoType Mycobacterium CM/AS assay are shown in Table 1. Five kinds of species/complex were identified, including *M*. *abscessus-M*. *immunogenum*, *M*. *intracellulare*, *M*. *Kansasii*, *M*. *peregrinum-M*. *alvei-M*. *septicum* and *Mycobacterium spec*. The percentages of each species are shown in Table 2. The most dominant NTM was *M*. *intracellulare* which accounted for 68.33% of the 60 isolates in the study. *M*. *abscessus-M*. *immunogenum* was the next most prevalent species (8 isolates, 13.33%), followed by *Mycobacterium spec*. (6 isolates, 10.00%), *M*. *Kansasii* (4 isolates, 6.67%) and *M*. *peregrinum-M*. *alvei-M*. *septicum* (1 isolate, 1.67%).

**Table 1:** Identification of mycobacteria by GenoType Mycobacterium CM/AS assay.

No. of strain	GenoType Mycobacterium CM/AS results	
	Band pattern*	Identification
1	1,2,3,9	*M*. *intracellulare*
2	1,2,3,9	*M*. *intracellulare*
3	1,2,3,9	*M*. *intracellulare*
4	1,2,3,9	*M*. *intracellulare*
5	1,2,3,9	*M*. *intracellulare*
6	1,2,3,9	*M*. *intracellulare*
7	1,2,3,9	*M*. *intracellulare*
8	1,2,3,10,12	*M*. *Kansasii*
9	1,2,3,9	*M*. *intracellulare*
10	1,2,3,9	*M*. *intracellulare*
11	1,2,3,9	*M*. *intracellulare*
12	1,2,3,5,6,10	*M*. *abscessus-M*. *immunogenum*
13	1,2,3,9	*M*. *intracellulare*
14	1,2,3,9	*M*. *intracellulare*
15	1,2,3,9	*M*. *intracellulare*
16	1,2,3,10	*Mycobacterium spec*.
17	1,2,3,9	*M*. *intracellulare*
18	1,2,3,9	*M*. *intracellulare*
19	1,2,3,9	*M*. *intracellulare*
20	1,2,3,9	*M*. *intracellulare*
21	1,2,3,5,6,10	*M*. *abscessus-M*. *immunogenum*
22	1,2,3,9	*M*. *intracellulare*
23	1,2,3,9	*M*. *intracellulare*
24	1,2,3,5,6,10	*M*. *abscessus-M*. *immunogenum*
25	1,2,3,10,12	*M*. *Kansasii*
26	1,2,3,9	*M*. *intracellulare*
27	1,2,3,9	*M*. *intracellulare*
28	1,2,3,9	*M*. *intracellulare*
29	1,2,3,9	*M*. *intracellulare*
30	1,2,3,9	*M*. *intracellulare*
31	1,2,3,9	*M*. *intracellulare*
32	1,2,3,9	*M*. *intracellulare*
33	1,2,3,9	*M*. *intracellulare*
34	1,2,3,10	*Mycobacterium spec*.
35	1,2,3,10	*Mycobacterium spec*.
36	1,2,3,9	*M*. *intracellulare*
37	1,2,3,10	*Mycobacterium spec*.
38	1,2,3,9	*M*. *intracellulare*
39	1,2,3,9	*M*. *intracellulare*
40	1,2,3,9	*M*. *intracellulare*
41	1,2,3,5,6,10	*M*. *abscessus-M*. *immunogenum*
42	1,2,3,9	*M*. *intracellulare*
43	1,2,3,9	*M*. *intracellulare*
44	1,2,3,9	*M*. *intracellulare*
45	1,2,3,10	*Mycobacterium spec*.
46	1,2,3,9	*M*. *intracellulare*
47	1,2,3,9	*M*. *intracellulare*
48	1,2,3,14	*M*. *peregrinum-M*. *alvei-M*. *septicum*
49	1,2,3,9	*M*. *intracellulare*
50	1,2,3,10,12	*M*. *Kansasii*
51	1,2,3,9	*M*. *intracellulare*
52	1,2,3,10,12	*M*. *Kansasii*
53	1,2,3,5,6,10	*M*. *abscessus-M*. *immunogenum*
54	*1*,*2*,*3*,*5*,*6*,*10*	*M*. *abscessus-M*. *immunogenum*
55	*1*,*2*,*3*,*5*,*6*,*10*	*M*. *abscessus-M*. *immunogenum*
56	1,2,3,10	*Mycobacterium spec*.
57	1,2,3,5,6,10	*M*. *abscessus-M*. *immunogenum*
58	1,2,3,9	*M*. *intracellulare*
59	1,2,3,9	*M*. *intracellulare*
60	1,2,3,9	*M*. *intracellulare*

*Identification of hybridization signals according to the instructions of the assay.

**Table 2:** The distributions of NTM identified by GenoType Mycobacterium CM/AS assay.

Mycobacterium species	Positive specimens	
	No.	Percent
*M*. *abscessus-M*. *mmunogenum*	8	13.33%
*M*. *intracellulare*	41	68.33%
*M*. *Kansasii*	4	6.67%
*M*. *peregrinum-M*. *alvei-M*. *septicum*	1	1.67%
*Mycobacterium spec*.	6	10.00%
Total	60	100.00%

**Fig 2:**
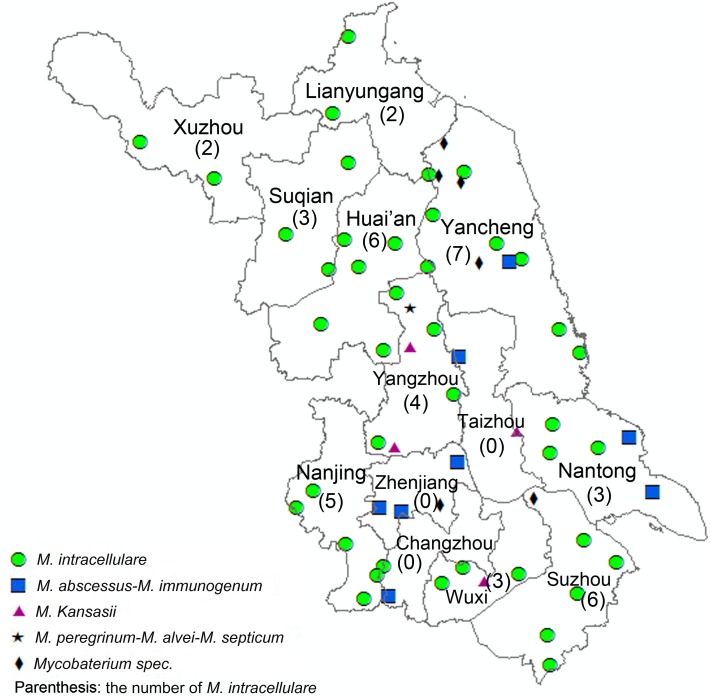
Geographical distribution of NTM in Jiangsu province. Data presented as number of *M*. *intracellulare* strains.

In order to investigate the geographical distribution and frequency of NTM, we plotted NTM distributions in 13 cities of Jiangsu province (Fig. 2). Except for in Changzhou, Taizhou and Zhenjiang, *M*. *intracellulare* had an extensive distribution throughout the province and was most frequent in Yancheng, followed by Huai’an and Suzhou (Fig. 2). Besides that, *M*. *abscessus-M*. *immunogenum* was present in five neighboring cities in the southeastern part of the province and *M*. *Kansasii* was only found in 3 cities located along the Yangzi River. Only one isolate of *M*. *peregrinum-M*. *alvei-M*. *septicum* was found in Yangzhou city. The members of *Mycobacterium spec*. was detected in three different cities, Yancheng, Suzhou and Zhenjiang, located in the eastern part of Jiangsu province.

## Discussion

Worldwide, pulmonary disease caused by NTM is increasing [10,20] and has captured more awareness and interest among the isolates of all species of mycobacteria. However, there is no evidence of direct transmission of NTM between humans. Due to this, NTM is not a notifiable condition in many countries and remains unmonitored by governmental agencies. Our study revealed that the overall proportion of NTM isolates from whole specimens was 3.37%, slightly lower than the mean rate in Shanghai, China [21] and much lower than reports from Europe [22]. We also elucidated the distributions of NTM species to analyze, for the first time, the geographical character in the eastern part of China. *M*. *intracellulare* was the dominant strain and was almost evenly distributed in this area. Meanwhile, *M*. *abscessus-M*. *immunogenum* and *M*. *Kansasii* were restricted to several adjacent cities of Jiangsu province. The distributions of NTM species varied by region and may have a profound impact on the prevalence of pulmonary NTM disease.

For *M*. *avium* complex (MAC), the most common NTM, infections in immunocompetent patients are principally pulmonary [3].The average treatment failure rates of MAC was as high as 20–40% [11]. Another work in Japan indicated that the overall mortality rate was 28.0% and the mortality for untreated MAC patients was 10% higher than for treated patients [12]. As a member of the MAC, *M*. *intracellulare* was the dominant strain in Jiangsu province in accordance with previous studies. In Korea, Jae Kyung Kim et al. found that *M*. *avium* complex was the most common NTM and *M*. *intracellulare* accounted for 51.3% of all specimens [23]. Other research conducted in east Asia showed that *M*. *avium* complex bacteria were also the most frequent isolates (13%–81%) and the most common cause of pulmonary NTM disease (43%–81%) [24]. In addition, *M*. *intracellulare* was the most frequently isolated strain in South Africa and Australia, [6]. The high frequency of *M*. *intracellulare* reported in different studies may be due to extensive distribution in the environment, especially in potable water [25]. When we focused on the geographical distribution of *M*. *intracellulare*, we found it almost evenly distributed in the province, although absent in three cities (Zhenjiang, Taizhou and Changzhou). The underlying causes of the absence seen in these cities is not clear, but the three cities located in the southern part of Jiangsu province have a relatively lower prevalence of *M*. *tuberculosis* [26]. Considering the samples were from tuberculosis suspicious patients, we presumed that the low epidemic situation of tuberculosis was one factor.

*M*. *intracellulare* has been reported in association with HIV infection [12] as well as with increasing frequency in the non-AIDS population [27]. Our study for the first time described the current situation of NTM caused lung disease and the species proportions in the eastern part of China, where a lower prevalence of HIV infection exists [28]. In our study, TB suspects are not high-risk populations for HIV infection, such as injecting drug users, and therefore HIV status was not detected for each subject.

The second most frequently isolated NTM in our study is *M*. *abscessus-M*. *immunogenum* complex. According to the interpretation chart provided by the CM/AS manufacturer, we couldn’t identify between these two species because they share the same line probe bands. Previous work suggested that *M*. *abscessus* was one of the most frequent species of rapid growers and usually concerned with skin, soft tissue and pulmonary infections [29]. As an example, it was the third most frequently isolated NTM species in Taiwan and the second most frequently isolated in South Korea [6]. *M*. *immunogeum* has been identified in metalworking fluids and has been shown to be highly correlated to hypersensitivity pneumonitis [30]. In our study, *M*. *abscessus-M*. *immunogenum* was found to be restricted to the southeastern part of Jiangsu province. *M*. *kansasii* often produces infiltrates or cavities in the upper lobes of immunocompetent patients [31]. In South America, *M*. *kansasii* was the second most isolated NTM after MAC, accounting for 19.8% of all NTM [6]. But in Jiangsu province it was not the prevalent species according to our study and showed very limited geographic spread.

We encountered the occasional presence of other rare species in this study, such as *M*. *peregrinum-M*. *alvei-M*. *septicum*. This species appeared only in one region and the isolate was too scarce to include in analysis however. Besides that, there were six specimens that failed with the CM/AS assay, only identifying as *Mycobacterium spec*. This may be due to the limited discriminative ability of this assay. Similar results have been seen in previous studies, where cross reaction among NTM species was supposed as the reason for the discrepant results [32,33].

Considering the rate of MDR TB in Jiangsu province is higher than the epidemiological situation of all of China [26], there was a high possibility of misdiagnosis of NTM in clinic. Usually, NTM is resistant to first-line anti-TB drugs, so misdiagnosis leading to inappropriate treatment can result in poor outcome. Our study could be beneficial for distinguishing NTM from M. tuberculosis and promoting valid clinical diagnoses of NTM.

According to the American Thoracic Society (ATS) document on NTM diagnosis [34], clinical symptoms and manifestation for TB suspicion in combination with laboratory identification increases diagnostic accuracy of Mycobacterium caused lung disease. However, several shortcomings of our study should be mentioned. Firstly, according to the criteria for subject inclusion, those subjects with pulmonary symptoms for less than 2 weeks would be ignored and the actual prevalence for NTM caused pulmonary disease would likely be underestimated. In addition, we did not follow-up patients therefore we could not analyze NTM treatment outcomes.

In summary, we performed a new reverse hybridization technique to illustrate the NTM species distribution from sputum specimens in the eastern region of China and established a procedure to identify and confirm NTM. Given the clinical challenge, further knowledge of the epidemiology of NTM in Jiangsu province is needed and the varying distribution of NTM species by region might have a profound and lasting impact on prevalence of pulmonary NTM disease. In addition, research efforts should be directed towards areas that will lead to strategies to prevent, predict, and improve treatment of NTM disease.

## Supporting Information

S1 Checklist STROBE Statement—Checklist of items that should be included in reports of *cross-sectional studies*. (DOC)
